# Oral antibiotic bowel decontamination in open and laparoscopic sigmoid resections for diverticular disease

**DOI:** 10.1007/s00384-021-03890-1

**Published:** 2021-02-19

**Authors:** Ulrich Wirth, Josefine Schardey, Thomas von Ahnen, Petra Zimmermann, Florian Kühn, Jens Werner, Hans Martin Schardey, Bettina M. Rau, Julia Gumpp

**Affiliations:** 1 Department of General, Visceral, and Transplant Surgery, Ludwig Maximilian University of Munich, Marchioninistr. 15, D-81377 Munich, Germany; 2 Department of General, Visceral, Vascular and Endocrine Surgery, Agatharied Hospital, Hausham, Germany; 3 Department of Surgery, Kliniken des Landkreises Neumarkt, Neumarkt, Germany

**Keywords:** Oral antibiotic decontamination, Anastomotic leakage, Oral antibiotics, Diverticulitis, Sigmoid resection, SDD

## Abstract

**Purpose:**

There is an ongoing debate on whether or not to use oral antibiotic bowel decontamination in colorectal surgery, despite the numerous different regimens in terms of antibiotic substances and duration of application. As we routinely use oral antibiotic bowel decontamination (selective decontamination of the digestive tract (SDD) regimen and SDD regimen plus vancomycin since 2016) in surgery for diverticular disease, our aim was to retrospectively analyze the perioperative outcome in two independent centers.

**Methods:**

Data from two centers with a routine use of oral antibiotic bowel decontamination for up to 20 years of experience were analyzed for the perioperative outcome of 384 patients undergoing surgery for diverticular disease.

**Results:**

Overall morbidity was 12.8%, overall mortality was 0.3%, the overall rate of anastomotic leakage (AL) was 1.0%, and surgical site infections (SSIs) were 5.5% and 7.8% of all infectious complications including urinary tract infections and pneumonia. No serious adverse events were related to use of oral antibiotic bowel decontamination. Most of the patients (93.8%) completed the perioperative regimen. Additional use of vancomycin to the SDD regimen did not show a further reduction of infectious complications, including SSI and AL.

**Conclusion:**

Oral antibiotic decontamination appears to be safe and effective with low rates of AL and infectious complications in surgery for diverticular disease.

## Introduction

Diverticular disease is a common burden in western world with increasing incidence over the past decades [1, 2]. Despite growing prevalence of asymptomatic diverticulosis with increasing age, only about 20% of affected people suffer from diverticulitis [1, 3]. In cases of chronic recurrent, acute, or chronic complicated cases, urgent or elective surgery can be necessary, not only to control a septic focus like abscess or fistula but also to avoid recurrence [1–4]. Laparoscopic technique and enhanced recovery protocols changed the perioperative management substantially, not only in elective but also in urgent or emergency cases [2, 5]. Yet treatment and especially surgical intervention for symptomatic diverticular disease should follow current guidelines [1, 2, 6–10], but there is some discrepancy between the 2018 expired German national guidelines and the recently published ASCRS and ESCP guidelines. Especially the indication for surgery in diverticular disease changed over the past years, but despite different options for classification of diverticular disease, the surgical indication should primarily depend on the risk for recurrence or a complicated course based on disease severity, which in Germany nowadays is classified according to the “Classification of Diverticular Disease” (CDD) [8–10]. Nevertheless, in elective surgery for diverticular disease, there is a relevant risk for surgical and nonsurgical complications even in the era of laparoscopic surgery and enhanced recovery concepts [5, 11–13].

In elective colorectal surgery, the concept of “selective decontamination of the digestive tract” (SDD) with use of topical antibiotics is current subject of debate among colorectal surgeons [14–17]. The role of bacteria in the development of anastomotic leakage (AL) and surgical site infections (SSIs) has been clarified over the past years, and the use of oral antibiotic bowel decontamination (OABD) [18–21] and combined OABD and mechanical bowel preparation (MBP) is widely recommended in elective colorectal surgery [14, 15, 22–24]. Despite that recommendation, especially in Europe, there is no widespread use of OABD regimens but an ongoing debate of its efficacy for prevention of AL and decreasing SSI or infectious complications in general [15]. Data from recent published randomized controlled trials are inconsistent due to essential differences in OABD regimens [18, 25–27].

Up to now, there are no studies concerning the use of OABD or antibiotic bowel decontamination in surgery for diverticular disease. Only a few cases of sigmoid resections are included in some of the randomized controlled trials, but they were not separately analyzed due to the small sample sizes [25, 27].

Based on the modified SDD regimen introduced by Schardey et al. in gastric and rectal cancer surgery [18, 28], the perioperative OABD has been used in colorectal surgery at the Surgical Department of Agatharied Hospital (AH) since 1999 [17] and was successfully introduced at the Surgical Department of Neumarkt Hospital (NH) as well.

Our aim is to analyze the safety and efficacy of routine use of an OABD regimen with regard to infectious complications like AL and SSI and adverse events related to medication in surgery for diverticular disease in two regional hospitals.

## Patients and Methods

### Patients

We performed a bicentric retrospective cohort study in two regional hospitals. The study was approved by the local review board. All elective procedures of open and mainly laparoscopic sigmoid colon resections for symptomatic or complicated diverticulitis using OABD based on prospectively collected data from the hospitals’ databases were analyzed. A total of *n* = 384 resections was performed, thereof 332 between 1999 and 2019 in AH and *n* = 52 between 2017 and 2019 in NH. Indication for sigmoid colonic resections in Germany were based in the past on Hansen and Stock classification, later on CDD classification [8, 29] and the German national guidelines [8]. According to current recommendations, surgery was performed electively in a noninflammatory state if possible, at least 4–6 weeks after the last episode of diverticulitis [1, 2, 8]. Only patients with primary anastomosis on treatment with OABD, who started the antibiotic regimen on the day before surgery, were included in this analysis.

### OABD protocol

The OABD regimen consisted of polymyxin B (100 mg), gentamicin (80 mg), and amphotericin B (500 mg) in *n* = 253 patients from AH between 1999 and 2015 (PG regimen); from 2016 to 2019 in both hospitals, a regimen consisting of PG plus vancomycin (125 mg) was used in *n* = 79 (AH) and *n* = 52 (NH) cases (PGV regimen) [18, 28]. OABD was administered orally in the majority of cases from the day before surgery until the 7th postoperative day, a total of 30 dosages per patient. The total costs for the OABD were EUR 105.60.- for PG and EUR 335.70.- for PGV regimen (3.52 Euro/11.19 Euro per dosage). In patients receiving a protective loop ileostomy, a Foley catheter was placed transanally during surgical procedure for the topical application of the OABD. Bowel preparation and perioperative management followed the principles of the enhanced recovery regimen using only a mild laxative (e.g., Prepacol®, Guerbet GmbH, Germany). No MBP was performed. For the details of perioperative regimen, see Table 1. A systemic antibiotic prophylaxis was administered just before surgery according to WHO guidelines [22] and evidence [30]. Only in few cases with local septic focus, mostly in urgent surgical cases, a prolonged systemic antibiotic therapy was administered (< 5%).

**Table 1:** Oral antibiotic regimen for bowel decontamination in two centers

	Center		
	AH		NH
Period of time	1999–2015	2016–2019	2017–2019
Patients (*n*)	253	79	52
Regimen	PG: Polymyxin B (100 mg) Gentamicin (80 mg) Amphotericin B (500 mg)	PGV: Polymyxin B (100 mg) Gentamicin (80 mg) Amphotericin B (500 mg) Vancomycin (125 mg)	
Application	Day before surgery	2 PG(V) at 6 p.m.	
	Day of surgery	1 PG(V) at 0 a.m.–6 a.m.–(12 a.m.)–6 p.m.	
	POD 1–6	1 PG(V) at 0 a.m.–6 a.m.–12 a.m.–6 p.m.	

AH: Agatharied Hospital; NH: Neumarkt Hospital; POD: postoperative day

### Surgical technique

The surgical procedures were performed according to the current technical standards of open and laparoscopic procedures by overall eight experienced colorectal surgeons (3 NH and 5 AH). The colon was divided in the upper third of the rectum with a linear stapler (Contour linear stapler Ethicon Endo-Surgery, Johnson and Johnson, USA). A circular double row stapler (ILS Circular Stapler, Ethicon Endo-Surgery, Johnson and Johnson, USA) was used for creating the anastomoses in both hospitals applying the double-stapling technique. Routinely, a leak test was performed using a methylene-blue solution, air, or intraoperative rectoscopy. Urgent surgery was defined as either a delayed emergency surgery for acute complicated diverticular disease/diverticular bleeding or as an early elective surgery because of failure of conservative therapy with the possibility for preoperative OABD starting the evening before the surgical procedure.

### Data management and variables

Perioperative data were collected together with demographic patient information in databases in both hospitals (Table 2). In some cases, the extent of surgery had to be expanded for atypical liver resections, biopsy or concomitant cholecystectomy, bowel resections due to involvement in the inflammatory process, and quite often urogenital resections due to entero-vaginal or entero-vesical fistulas in chronic complicated diverticular disease.

**Table 2:** Data items included in analysis

Patient demographic data	Age	
	Sex	
	ASA score	
Perioperative data	Diagnosis according to CDD classification	
	OABD regimen and completeness	
	Surgical technique	
	Extension of surgery	Liver
		Bowel
		Urogenital
	Postoperative in-hospital stay	
	Diverting ileostomy	
	Time to ileostomy reversal	
Perioperative complications (≤ 30 days after surgery)	Anastomotic leakage	
	Surgical site infections	
	Wound hematoma	
	Pneumonia	
	Urinary tract infections	
	Cardiovascular complications	
	Other general complications	
	Hospital readmission	

OABD: oral antibiotic bowel decontamination; ASA: American Society of Anesthesiologists; CDD: Classification of Diverticular Disease.

Primary outcome measure is the rate of AL; diagnosis of AL was made by endoscopy, CT scan, or relaparotomy. Only cases of clinically apparent AL were recorded in our study. Furthermore, rates of SSI, pneumonia, urinary tract infections, cardiovascular complications, overall morbidity and mortality, and any adverse events related to OABD are analyzed. All surgical complications occurring within 30 days after surgical procedure were classified according to the Clavien–Dindo classification [31]. There is no control group without use of OABD available, since OABD is standard treatment in both centers and no elective or urgent sigmoid resections were performed without OABD.

### Statistical analysis

For statistical analysis SPSS Statistics 26 was used (IBM, Armonk, USA). The prospective databases were based on MS Excel (Microsoft Corporation, Redmond, Washington, USA). Descriptive statistics and calculation of the mean values were used to summarize patients’ characteristics and perioperative data. For comparison between subgroups, we used Kruskal–Wallis (KW) test for not normal-distributed values and independent samples. Normal distribution of the differences of the means was tested using the Shapiro–Wilk test. To compare nominal or categorical data *χ*^2^ and Fisher’s exact (FE) tests were used. *P* values < 0.05 were considered statistically significant.

## Results

A total of 384 patients undergoing sigmoid resection with primary anastomosis for diverticular disease were included. Most of the patients underwent surgery for chronic recurrent (CDD 3B: 60.7%), chronic complicated (CDD 3C: 19.5%), and acute complicated diverticulitis with macroabscess (CDD 2B: 14.0%). Most of the surgical procedures were performed electively (*n* = 359; 93.5%), only 25 patients (6.5%) had urgent procedures; 1 (0.3%) for acute diverticular bleeding, 12 (3.1%) for acute complicated diverticulitis (CDD 2A/B/C) without chance to postpone surgery, and another 12 (3.1%) for chronic recurrent/complicated diverticulitis with failure of conservative therapy. In only 1.0% (*n* = 4) of the cases, a protective loop ileostomy was created; 93.5% of surgical procedures were started laparoscopic, only 7 (1.8%) needed conversion to open surgery; the other 25 procedures (6.5%) were performed in conventional open surgical technique. Mean in-hospital stay after surgery was 8.8 days. For patient demographic data, see Table 3. All protective loop ileostomies could be reversed within 90 days after sigmoid resection (mean: 81.5 days).

**Table 3:** Patients’ clinical characteristics

*N*			384
Age (years)			61.9 ± 12.2
sex (male/female)			176/208
Postoperative in-hospital stay (days)			8.8 ± 5.5
Protective ileostomy			4 (1.0%)
Time to ileostomy reversal (days)			81.5 ± 18.5
Surgical technique	Open		25 (6.5%)
	Laparoscopic		352 (91.7%)
	Conversion		7 (1.8%)
OABD	Complete		360 (93.8%)
	Incomplete		24 (6.2%)
ASA score	I		65 (16.9%)
	II		241 (62.8%)
	III		77(20.1%)
	IV		1 (0.3%)
	V		0
CDD classification	0		0
	1	A	0
		B	1 (0.3%)
	2	A	11 (2.9%)
		B	54 (14.1%)
		C	2 (0.5%)
	3	A	1 (0.3%)
		B	233 (60.7%)
		C	75 (19.5%)
	4		7 (1.8%)

OABD: oral antibiotic bowel decontamination; ASA: American Society of Anesthesiologists; CDD: Classification of Diverticular Disease

The overall morbidity was 12.8% (*n* = 49). Only 1 patient who underwent urgent surgery for diverticular bleeding suffered a stroke and died in further clinical course (0.3%); no surgical complications occurred in this case. Overall, 30 patients (7.8%) developed infectious complication such as 21 (5.5%) SSI and 4 (1.0%) AL (Table 4). All cases of AL occurred in elective surgical procedures, 3 in laparoscopic cases and 1 in a case converted to open surgery. All AL required reoperation with discontinuity resection. In 3 of 4 cases, bowel continuity could be restored by a mean of 151.3 days after initial sigmoid resection. Wound hematoma occurred in 2.6% of cases. No clostridium difficile infections occurred. In 16 of 21 cases with SSI or AL (76%) and only 2 of 10 cases with urinary tract infection or pneumonia microbiologic analysis detected specific germs: *Enterococcus* species in 7 cases, *Staphylococcus* species in 8 cases (only 1 case with methicillin-resistant *Staphylococcus aureus*), *Pseudomonas aeruginosa* in 2 cases and candida species in 2 cases, and *Escherichia coli* in 1 case. Table 4 gives an overview on all perioperative complications and their classification.

**Table 4:** Perioperative complications

Morbidity			49 (12.8%)
Mortality			1 (0.3%)
SSI	Superficial		18 (4.7%)
	Deep		0
	Organ space		3 (0.8%)
	Total		21 (5.5%)
Anastomotic leakage	All		4 (1.0%)
	Grade A		0
	Grade B		0
	Grade C		4
	Ostomy reversal after anastomotic leakage		3/4 (75%)
	Time to terminal colostomy reversal		151.3 ± 69.1 days
Wound hematoma/seroma			10 (2.9%)
Bleeding from anastomosis with interventional treatment			1 (0.3%)
Ileostomy complication with need for surgical revision			1 (0.3%)
Incisional hernia			2 (0.5%)
Pneumonia			4 (1.0%)
Urinary tract infections			6 (1.6%)
Clostridium difficile infections			0
All infectious complications			30 (7.8%)
Stroke			2 (0.5%)
Myocardial infarction			3 (0.8%)
Clavien–Dindo classification	0		334 (87.0%)
	1		11 (2.9%)
	2		9 (2.3%)
	3	A	4 (1.0%)
		B	23 (6.0%)
	4	A	1 (0.3%)
		B	1 (0.3%)
	5		1 (0.3%)
Surgical reinterventions	*N* patients		25
	*N* total		58
Interventional therapy	*N* patients		6
	*N* total		8

SSI: surgical site infection

There was a substantial difference regarding the need for an extension of surgery onto other organs, mostly the urogenital tract due to fistulas (*n* = 23), between laparoscopic and open procedures (*χ*^2^; *p* = 0.005). Only 7.7% of the laparoscopic cases were associated with extension of surgery vs. 16.0% of cases carried out in open technique and 42.9% of cases with a need of conversion to open surgery. Furthermore, the distribution of age (KW; *p* = 0.005), duration of postoperative in-hospital stay (KW; *p* < 0.001), surgical complications in general (*χ*^2^; *p* < 0.001), SSIs (*χ*^2^; *p* < 0.001), urinary tract infections (*χ*^2^; *p* < 0.001), cardiovascular complications (*χ*^2^; *p* = 0.009), and all infectious complications were different (*χ*^2^; *p* = 0.005) for each surgical technique applied (Table 5).

**Table 5:** Differences between groups for surgical technique

		Laparoscopic	Open	Conversion	Total
*n*		352	25	7	384
Age (years)		61.3 ± 12.0	68.7 ± 13.0	64.7 ± 10.5	61.9 ± 12.2
Duration of postoperative in-hospital stay (days)		8.3 ± 4.8	13.8 ± 7.1	17.9 ± 12.2	8.8 ± 5.5
Protective ileostomy		3 (0.9%)	1 (4%)	0	4 (1.0%)
Multivisceral resections	Liver	4 (1.1%)	0	0	4 (1.0%)
	Bowel	3 (0.9%)	0	0	3 (0.8%)
	Urogenital	17 (4.8%)	3 (12.0%)	3 (42.9%)	23 (6.0%)
	Others	3 (0.9%)	1 (4%)	1 (14.3%)	4 (1.0%)
Anastomotic leakage		3 (0.9%)	0	1 (14.3%)	4 (1.0%)
Surgical complications		28 (8.0%)	3 (12%)	4 (57.1%)	35 (9.1%)
SSI	Superficial	13 (3.7%)	2 (8%)	3 (42.9%)	18 (4.7%)
	Deep	0	0	0	0
	Organ space	2 (0.6%)	0	1 (14.3%)	3 (0.8%)
Wound hematoma/seroma		9 (2.6%)	1 (4%)	0	9 (2.5%)
Pneumonia		4 (1.1%)	0	0	4 (1.0%)
Urinary tract infection		3 (0.9%)	2 (8%)	1 (14.3%)	6 (1.6%)
Infectious complications		22 (6.3%)	4 (16%)	4 (57.1%)	30 (7.8%)
Cardiovascular complications		3 (0.9%)	2 (8%)	0	5 (1.3%)
Mortality		1 (0.3%)	0	0	1 (0.3%)

SSI: surgical site infection

**Fig. 1:**
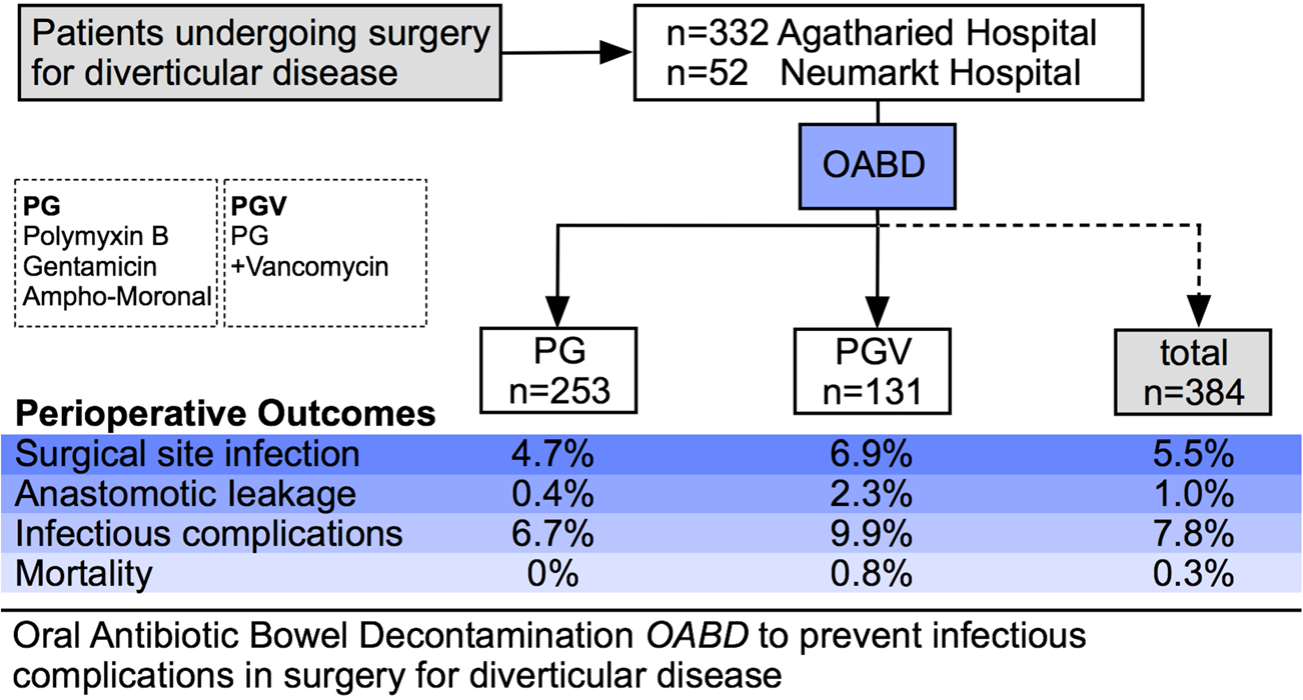
Summary of results and comparison of perioperative outcome for PG and PGV OABD regimens. OABD: oral antibiotic bowel decontamination; PG: polymyxin B + gentamicin + amphotericin B; PGV: polymyxin B + gentamicin + amphotericin B + vancomycin; SSI: surgical site infection

For the two antibiotic regimens used for OABD (PG vs. PGV), there was no difference between groups regarding AL (FE; *p* = 0.117), surgical complications (FE; *p* = 0.138), SSI (*χ*^2^; *p* = 0.639), rate of pneumonia (FE; *p* = 0.185) or urinary tract infections (FE; *p* = 1.0), infectious complications in general (FE; *p* = 0.316), mortality (FE; *p* = 0.341), and duration of perioperative application (FE; *p* = 1.0). Only the length of postoperative in-hospital stay was shorter in patients who received PGV compared to PG (KW; *p* = 0.024) (Table 6 and Fig. 1).

**Table 6:** Use of OABD: differences between groups for OABD regimen (PG vs. PGV)

		PG	PGV	Total
*n*		253	131	384
Duration of postoperative in-hospital stay (days)		9.35 ± 6.1	7.8 ± 3.8	8.8 ± 5.5
Completeness of OABD		237 (93.7%)	123 (93.9%)	360 (93.8%)
Reasons for OABD termination	PostOP ileus			11 (2.9%)
	Intolerance			7 (2.1%)
	Patient decision			3 (0.8%)
	Diarrhea			2 (0.5%)
Time to termination (mean, postoperative days)				3.3 ± 1.2
Protective ostomy		1	3	4
Surgical technique	Laparoscopic	225 (88.9%)	127 (96.9%)	352 (91.7%)
	Open	23 (9.1%)	2 (1.5%)	25 (6.5%)
	Conversion	5 (2.0%)	2 (1.5%)	7 (1.8%)
Anastomotic leakage		1 (0.4%)	3 (2.3%)	4 (1.0%)
Surgical complications		19 (7.5%)	16 (12.2%)	35 (9.1%)
SSI	Superficial	10 (3.9%)	8 (6.1%)	18 (4.7%)
	Deep	0	0	0
	Organ space	2 (0.8%)	1 (0.8%)	3 (0.8%)
Pneumonia		1 (0.4%)	3 (2.3%)	4 (1.0%)
Urinary tract infection		4	2	6 (1.6%)
Infectious complications		17 (6.7%)	13 (9.9)	30 (7.8%)
Clostridium difficile infections		0	0	0
Mortality		0	1 (0.8%)	1 (0.3%)

OABD: oral antibiotic bowel decontamination; PG: polymyxin B + gentamicin + amphotericin B; PGV: polymyxin B + gentamicin + amphotericin B + vancomycin; SSI: surgical site infection

The OABD was completed and well tolerated by most of our patients and therefore completed until 7th postoperative day (*n* = 360; 93.8%). Only 24 patients (6.3%) discontinued the antibiotic medication prematurely for different reasons and possible side effects; 11 (2.9%) patients had prolonged postoperative ileus with nausea and vomiting, and another seven (1.8%) had selective nausea after intake of medication, which was interpreted as an intolerance; in three cases (0.8%), medication was stopped by request of patients, and in two cases (0.6%), there was diarrhea without detection of clostridium difficile in the stool samples (Table 6). No other side effects or allergic reactions to the OABD regimen were observed.

## Discussion

We present the first clinical results of the routine use of OABD in surgery for diverticular disease with low AL, SSI, and mortality rates. Not only elective but also urgent surgical cases with bleeding and septic abdominal conditions are included. With these data, we also prove the feasibility of a perioperative regimen of OABD in routine clinical use.

In our bicentric analysis, we found low rates of AL (1%), SSI (5.5%), pneumonia (1%), urinary tract infections, (1.6%) and infectious complications in general (7.8%). Under OABD, all complication rates were low when compared to the reported rates achieved without this prophylaxis. In recent clinical trials and retrospective analyses in early and delayed surgery for diverticulitis, minor and major complication rates of about 7.5–36.0% and 2.2–9.6% are described [11, 12, 32]. Klarenbeek et al. report of SSI rates of 15.4% and AL rates of 5.8% in laparoscopic sigmoid resections [32]. A recently published study reported an outcome of 21% for SSI and 3.1% for AL in 1737 surgical cases of diverticulitis [33]. A retrospectively analyzed series on 168 surgical cases reported an outcome of 6.5% for AL and 10.8% for SSI in mostly delayed elective procedures and 15% urgent or emergency cases [6]; another retrospective Swiss cohort reports SSI and AL rates of 7.2% and 5.5% [34]. Overall, in the sparse available data the reported rates for SSI and AL vary between 5.2–21.0% and 1.8–9.2%, respectively; for other infectious complications like pneumonia and urinary tract infections, rates of about 1.5–4.1% and 4.6–5.8% are reported [6, 12, 13, 32–35]. Data report a low mortality between 0% and 2.2% mostly caused by septic complications and AL [11–13, 32–34]. We observed a mortality of only 0.3% as one patient died without surgical complications due to fatal stroke after emergency laparoscopic sigmoid resection for diverticular bleeding.

The AL rate of 1% with 3 laparoscopic and one converted case was low. We observed a trend of more surgical complications and SSI in the small number of converted cases (*n* = 7) compared to primary open and completely laparoscopically performed sigmoid resections. There were only a few patients in need for urgent sigmoid resections (6.5%) to treat acute or chronic complicated diverticulitis with a higher rate of open procedures (36%). The PG patients were treated between 1999 and 2015, whereas the PGV group was treated between 2016 and 2019. In-hospital stays shortened over the years, which explains the shorter hospital stay of the latter group. For the same reason, there were more patients with laparoscopic vs. open procedures in the PGV compared to the PG group. But an overall high rate of 91.7% of surgical procedures performed laparoscopically for diverticular disease (88.9% PG and 98.9% in PGV group) and insofar a technical homogenous patient collective, however, allows valid conclusions from this analysis despite the time period of 20 years.

The low number of infectious complications and especially low rates of SSI and AL in our series are consistent with other published data using different regimens of OABD in colorectal surgery [25]. Along with a decrease of SSI and AL, a better outcome regarding postoperative ileus and mortality is reported [18, 23, 30]. Schardey et al. demonstrated a fourfold decrease in rates of AL and nearly 50% decrease for infectious complications in rectal cancer surgery [18]. The recently published SELECT trial however did not show a significant reduction in AL (9.7% control and 6.1% in SDD group) but a reduction of nearly 50% (26.9% to 14.9%) of all infectious complications [25]. In a very comprehensive meta-analysis by Rollins et al., OABD was found to be associated with a relevant reduction of SSI (RR 0.51; 0.46–0.51) and AL (0.62; 0.55–0.70) in the included registry data [23]; the reduction of AL however was not detected in the reviewed RCTs (RR 0.69; 0.43–1.11; *p* = 0.13) [23]. At the same time, the available studies are inconsistent in terms of the regimen of antibiotic drugs, duration of OABD, and the type of gastrointestinal surgery [18, 23, 25–27, 30]. Overall, the use of topical antibiotics based on SDD regimens [18, 24, 25, 27] was highly effective in the reduction of SSI, AL, and other infectious complications in contrast to other concepts [26]. We recently reported on a retrospective analyzed series on patients with rectal cancer surgery and topic antibiotic bowel decontamination with similarly low rates of AL (5.8%) and SSI (19.9%) [17]. In summary, the available data confirm an advantage of the use of topically applied antibiotics in colorectal surgery compared to controls regarding infectious complications, especially SSI. The results concerning the prevention of AL are more inconsistent but nonetheless are positive in studies, when antibiotic regimens are used in a reasoned combination [17, 18, 23–25, 27, 30].

There has been a growing evidence for more than 60 years that bacteria play a central role in the pathogenesis of anastomotic leak. In an experimental setting, topical antibiotics were shown to prevent AL even in the presence of severe ischemia, while systemic antibiotics had no protective effect [36].

In recent years, Alverdy and coworkers unraveled several of the molecular mechanisms used by some bacteria to break down anastomotic tissue [20, 21, 37, 38]. Central to the pathophysiology of AL seems to be the high collagen degradation activity of these microorganisms [37] turning on their virulence with collagenase production or the activation of MMP9 or plasminogen [37, 38]. The breakdown of collagen causes anastomoses to lose their mechanical stability and inflammation proceeds [20, 21, 37]. In experimental settings, *Enterococci*, *Pseudomonas*, and *Serratia marcescens* are pathogens associate with AL [21, 37, 38].

Therefore, the concept behind OABD is the elimination of potentially pathogenic bacteria from the gastrointestinal tract thereby also eliminating bacterial virulence factors [39]. Because of the apparent role of enterococci species [37, 38], we changed our use of topical antibiotics from PG to the originally used PGV regimen [18, 28]. In the present analysis, most of the infections were caused by gram-positive cocci or *Pseudomonas aeruginosa*. Nevertheless, our SDD-based concept of OABD with combination of gentamicin and polymyxin covers gram-negative pathogens twice with the exception of proteus since polymyxin has a gap in this regard, while gentamicin and vancomycin both covers gram-positive pathogens. Amphotericin B protected from fungal overgrowth. We did not observe any negative side effects like clostridium difficile enteritis or antibiotic resistance. SSI and AL, overall morbidity, and mortality rates were very low in this series just as in our previously published RCTs of gastric and rectal surgery using this OABD regimen [17, 18]. Previous analysis demonstrated that the use of OABD regimen is cost-effective despite the additional costs of about 105 Euro for PG and 335 Euro for PGV regimen and could decrease the treatment costs by about 19% in gastric cancer surgery and even 38% in rectal cancer surgery due to the decrease of days of in-hospital stay, days on intensive care units, and number of surgical or interventional procedures [18, 40]. According to these data and a recently published analysis by Bordeianou et al., infectious complications are frequent and costly complications and therefore strategies to prevent these complications are urgently necessary in colorectal surgery [18, 33, 40]. However, as we were unable to demonstrate a benefit from the additional use of vancomycin in the surgical cases presented here and taken together with the much higher treatment costs for the PGV compared to the PG regimen, the PG regimen without vancomycin might be sufficient to relevantly decrease the rates of infectious complications based on our data.

The use of OABD deserves a critical look especially in times of increasing rates of multidrug-resistant germs [41]. However, the available data on the routine use of topical antibiotics like SDD regimen in intensive care units only demonstrate a decrease of colonization, e.g., with enterococci species [42, 43]. Nonetheless, as the load of multidrug-resistant germs increases, the analysis of microbiome signatures might be favorable [20, 21, 38, 39], in order to tailor the composition of an OABD for the individual patient. Ideal however would be the avoidance of antibiotics altogether in the future. Alverdy et al. already have investigated different nonantibiotic, antivirulence agents to prevent AL, which should be tested in clinical trials rather sooner than later [39].

The study is limited by its retrospective character and missing control group. Due to the retrospective character an underestimation of some minor complications may be possible, as some superficial SSI may have been occurred after hospital discharge without readmission to the hospital. Especially for relevant complications and major morbidity, this bias can be assumed to be not relevant, as they mostly occur during the in-hospital stay or patients would have been readmitted to the regional hospitals for treatment of such complications. Furthermore, we compare our data to some other available retrospective or registry series, and therefore, the same bias can be assumed in these data [6, 33, 34].

The data are obtained over a 20-year time period, and therefore, some issues like in-hospital stay after surgery of 8 days may not be estimated adequate anymore. As mentioned above, our OABD regimen ends on the 6th postoperative day, and our present research is focusing on using less topical antibiotics after surgery for shorter periods of time allowing for an earlier hospital discharge. Nevertheless, other even recently published data report of in-hospital stays about 7–13 days [6, 33, 34].

The treatment during the included period of time followed national guidelines and evidence, but these guidelines changed over the years, and right now, the German national guidelines for the diagnosis and treatment of diverticular disease are expired [8]. Recently, new American and European guidelines have been published, and as our knowledge about the risk of recurrence and complicated course in diverticular disease further increased, the indication for surgery has also changed over the past decade. Therefore, there are some surgical cases included in our analysis, which might not be recommended for sigmoid resections according to these guidelines anymore [9, 10]. Besides, there are a bunch of classifications for diverticular disease and acute diverticulitis, and as none of these classifications seems to be better validated than the others, we use the CDD classification according to the German guidelines [8–10]. This CDD classification is thereby based on the former used Hinchey and Hansen/Stock classifications but additionally includes diagnostic and therapeutic aspects [8]. Overall, despite these limitations, our analysis represents surgical everyday life as our standard of care very well.

## Conclusion

According to our results, OABD seems to be an effective and safe tool for prevention of not only AL but also SSI and other infectious complications in the surgery of diverticular disease. A decrease of these infectious complications in gastrointestinal surgery can not only decrease additional treatment costs but also improve our patients’ quality of life and health, which should be the primary focus of our work as surgeons [33].

## Data Availability

Because of the content of personal data, the data and material are not available in public.
